# GC‐MS metabolite profiling for specific detection of dwarf somaclonal variation in banana plants

**DOI:** 10.1002/aps3.1194

**Published:** 2018-11-12

**Authors:** Juan M. Cevallos‐Cevallos, Cristina Jines, María G. Maridueña‐Zavala, María J. Molina‐Miranda, Daniel E. Ochoa, José A. Flores‐Cedeno

**Affiliations:** ^1^ Centro de Investigaciones Biotecnológicas del Ecuador Escuela Superior Politécnica del Litoral, ESPOL Campus Gustavo Galindo Km 30.5 Vía Perimetral, P.O. Box 09‐01‐5863 Guayaquil Ecuador; ^2^ Facultad de Ciencias de la Vida Escuela Superior Politécnica del Litoral, ESPOL Campus Gustavo Galindo Km 30.5 Vía Perimetral, P.O. Box 09‐01‐5863 Guayaquil Ecuador; ^3^ Facultad de Ingeniería Eléctrica y Computación Escuela Superior Politécnica del Litoral, ESPOL Campus Gustavo Galindo Km 30.5 Vía Perimetral, P.O. Box 09‐01‐5863 Guayaquil Ecuador; ^4^ Present address: Facultad de Farmacia Departamento de Biología Vegetal Universidad de Valencia 46100 Valencia Spain

**Keywords:** cucumber mosaic virus, metabolomics, *Musa*, Musaceae, partial least squares regression, somaclonal variation

## Abstract

**Premise of the Study:**

The production of banana (*Musa* spp.; Musaceae) plants is affected by various types of somaclonal variations (SV), including dwarfism. However, methods for specific detection of SV are still scarce. To overcome this, a metabolite‐based method for detection of dwarf variants was evaluated.

**Methods:**

The gas chromatography–mass spectrometry (GC‐MS) metabolite profile of dwarf banana variants was investigated and compared to that of normal‐healthy (N) and cucumber mosaic virus (CMV)–infected plants using principal components analysis and partial least squares discriminant analysis (PLS‐DA).

**Results:**

Significant differences among the sample groups were observed in 82 metabolites. Rhamnose was exclusively present in dwarf plants but allothreonine and trehalose were present in all but SV samples. Cellobiose was only detected in N plants, while 45 other metabolites, including methyl‐glucopyranoside, allopyranose, lactose, phenylalanine, and l‐lysine were detected in all but CMV‐infected samples. PLS‐DA models were able to detect SV, CMV, and N plants with 100% accuracy and specificity.

**Discussion:**

The GC‐MS metabolite profile can be used for the rapid, specific detection of SV at early plant production stages. This is the first metabolite‐based characterization and detection of somaclonal variation in plants.

Metabolomics—the study of small metabolites in a system—has been successfully applied in the plant sciences to investigate plant development (Watanabe et al., 2013; Hu et al., 2016), characterize plant response to abiotic stresses (Caldana et al., 2011; Maruyama et al., 2014), and diagnosis plant diseases (Cevallos‐Cevallos et al., 2009, 2011). Among various metabolomics tools, the use of gas chromatography–mass spectrometry (GC‐MS) has been recommended for the assessment of plant varieties with different morphologies (Cevallos‐Cevallos et al., 2012), because metabolites have shown a more relevant relationship to plant phenotypes than genes (Fiehn, 2001; Yogendra et al., 2014). Therefore, metabolomics tools have the potential for characterizing morphological alterations in plants and detecting somaclonal variants.

Banana (*Musa* L. spp.; Musaceae) is one of the most cultivated crops worldwide and a major source of income for millions of people in many countries (Yeturu et al., 2016). The world's total production of banana was estimated at 113 million tons in 2016, with Ecuador standing out as the top banana‐exporting country (Food and Agriculture Organization of the United Nations, 2017). Banana plants are commonly produced by in vitro micropropagation techniques focusing on the generation of phenotypically uniform plantlets from a parent plant. However, phenotypic variations—known as somaclonal variations (SV)—are commonly observed in about 6% to 69% of the *Musa* spp. plants regenerated by tissue culture (Sahijram et al., 2003). Various types of atypical plant morphologies have been associated with SV, with dwarfism being the most common SV observed (Reuveni and Israeli, 1990).

Plants showing SV are commonly considered to be of inferior quality and reduced commercial value when compared to the parental clones (Sahijram et al., 2003; Oh et al., 2007), but improved characters of somaclonal variants have also been reported (Bairu et al., 2010). Dwarf variants of the ‘Williams’ banana cultivar can bear shorter fruits of atypical shapes (Israeli et al., 1991) but can show faster growth and flowering rates (Israeli et al., 1991) than normal plants. Additionally, the stocky build of dwarf bananas suggests a higher resistance to physical damages and advantages of cultivation convenience, field management, and labor savings when compared to normal clones (Ferrero‐Serrano and Assmann, 2016).

Detection of SV in *Musa* spp. has traditionally been carried out through visual examination of the plants. Dwarf off‐types have been detected by observing the plant height, leaf index (leaf length/width; Rodrigues et al., 1998), and pseudostem thickness (Oh et al., 2007) at the greenhouse production stage. However, banana morphological traits can also be affected by environmental factors (Bairu et al., 2010) and biotic stresses such as cucumber mosaic virus (CMV) infections (Yeturu et al., 2016), rendering visual diagnosis of SV unreliable. CMV can be transmitted by more than 80 aphid species and infect almost 1200 plant species including bananas (Zitter and Murphy, 2009), making CMV one of the most widespread banana viruses worldwide (Basavaraj et al., 2017). CMV can be detected wherever bananas are grown (Dheepa and Paranjothi, 2010), and *Musa* spp. plants infected by CMV can show symptoms resembling those of SV, including growth deficiency and leaf distortion (Yeturu et al., 2016), leading to problems of misdiagnosis between CMV and SV. Therefore, non‐visual methodologies need to be applied for the detection of SV in *Musa* spp. at early plant production stages, and the specificity of SV diagnosis methods must be validated against CMV‐infected plants.

Various DNA‐based methodologies have been proposed to assess genetic stability as a means to infer SV in micropropagated banana plants, including random‐amplified polymorphic DNA (RAPD) (Sheidai et al., 2008), inter‐simple sequence repeat (ISSR) (Ray et al., 2006), amplified fragment length polymorphism (AFLP) (Sahijram et al., 2003), and representational difference analysis (RDA) (Oh et al., 2007), among others. However, SV in clones showing polymorphic DNA was not phenotypically confirmed, and no association between DNA markers and alterations of visual traits was made.

Biochemical methods have also been suggested to detect dwarf banana variants, including the quantification of gibberellic acids (GAs) (Chen et al., 2016). However, GA is not the only endogenous signal that induces plant growth, and GA‐unresponsive dwarf variants have been reported (Sun, 2000). Moreover, the specificity of biochemical tests against CMV‐infected plants has not been evaluated. Specificity of biochemical methods for detection of various plant conditions can be significantly improved using a group of metabolites instead of a single marker (Cevallos‐Cevallos et al., 2009), but no metabolomics‐based characterization of banana dwarf variants can be found in the literature. Despite the importance of SV to the banana industry, reliable methods for the early and specific detection of SV in *Musa* spp. are still unavailable.

This research aimed to describe the GC‐MS metabolite profile of normal, dwarf, and CMV‐affected banana plants at the greenhouse establishment stage and propose a metabolomics‐based method for detection of dwarf variants.

## METHODS

### Plant material

Plants of the ‘Williams’ cultivar were obtained from a commercial banana propagation facility in Guayaquil, Ecuador, during 2015 and 2016. Plants in the greenhouse establishment phase (3–4 months) propagated from the same parental clone were selected for this study as suggested in previous reports (Rodrigues et al., 1998; Bairu et al., 2010). A total of 20 SV (dwarf variants), 16 normal‐healthy (N), and 15 CMV‐infected plants were collected. Only clones showing significantly shorter heights and smaller leaf indexes than standard normal plants were considered as dwarf variants (Israeli et al., 1991), whereas plants showing CMV symptoms, including yellow stripes on leaves, leaf distortion, and stunting of growth were preliminarily selected as putative CMV‐infected plants. The presence of CMV was then confirmed using a commercial triple antibody sandwich ELISA kit (Agdia, Elkhart, Indiana, USA) and CMV‐positive samples were selected for the study. The presence of CMV was also assessed in the SV and N samples yielding negative ELISA results. Plants were transported to our research facilities and kept at 28°C, 70% relative humidity with natural light (12 h) in a greenhouse, and watered every 48 h until analyzed (2 weeks).

### Metabolite analysis

For metabolite characterization, small (5 × 5 cm) pieces were simultaneously extracted from the center of three different leaves from each plant using a scalpel while avoiding the midrib. Metabolite extraction, separation, and detection were executed on each leaf piece as described elsewhere (Cevallos‐Cevallos et al., 2011, 2012). Briefly, each leaf piece was ground under liquid nitrogen and 800 mg were mixed with 2 mL of an 8 : 1 : 1 methanol : water : chloroform solution followed by incubation at 7°C for 48 h. Extracts were then centrifuged at 21130 *× g* for 2 min using an Eppendorf 5424 microcentrifuge (Hamburg, Germany) and the pellet was discarded. Aliquots of 650 μL of each extract were transferred into 2‐mL vials and incubated with the cap open in a water bath at 50°C until dry. A total of 150 μL of *N*‐methyl‐*N*‐trimethylsilyltrifluoroacetamide (MSTFA) was added to the dried samples and incubated at 85°C for 90 min. Different amounts of MSTFA, different incubation temperatures, and different reaction times were tested but yielded lower numbers of detected peaks and poorer reproducibility, as reported in previous studies (Cevallos‐Cevallos et al., 2011) and validated in this research. The solution (1 μL) was then splitlessly injected into a GC‐MS. The injector was at 250°C, the initial oven temperature was 80°C held for 1 min, the temperature rate was 7°C/min, and the final temperature of 300°C was held for 5 min. Ultrapure helium was used as the carrier gas at 1 mL/min. The GC‐MS interface was set to 280°C, and after 8 min of solvent delay the scan was recorded with a frequency of 4 Hz. Data were acquired using ChemStation E.02.02 software (Agilent Technologies, Santa Clara, California, USA), and differentially expressed metabolites were putatively identified by MS spectra matching using two databases: the National Institute of Standards and Technology (NIST) Mass Spectral Library (NIST 11) and the Wiley Registry of Mass Spectral Data, 9th ed. (Wiley 9) (McLafferty, 2009). Metabolite identity was then confirmed by comparing the linear retention index of each compound with that of the pure standard using our internal database. All metabolites were quantified by estimating the peak area using the ChemStation software as suggested for untargeted analysis (Maridueña‐Zavala et al., 2017; Cevallos‐Cevallos et al., 2018). Preliminary GC‐MS runs showed that peaks were too close to each other throughout the chromatogram and the use of internal standards would have interfered with the chromatographic peaks. For this reason, normalization and quality control (QC) techniques not relying on internal standards were applied, including normalization to the total area (Cevallos‐Cevallos et al., 2012; Wu and Li, 2016) and QC by running the same sample after specific intervals (Warth et al., 2015; Maridueña‐Zavala et al., 2017). The quality of the GC‐MS runs was assessed by running one selected extract every five runs and estimating the variations in retention time and peak areas. Maximum acceptable coefficient of variation was 30% for a given metabolite in QC runs (Warth et al., 2015; Maridueña‐Zavala et al., 2017). Three replicates were run for each analysis.

### Data analysis

Peak areas of each metabolite were used for data analysis. Peaks detected in less than one of six measurements were marked as potential metabolite carry‐over and were excluded from the analysis (Warth et al., 2015). Data were normalized to the total area and aligned using in‐house protocols based on metabolite comparisons of the MS spectra and retention times (Cevallos‐Cevallos et al., 2011). Differentially expressed metabolites were selected based on Student's *t*‐test comparisons of SV or CMV against N plants at the 0.05 significance level and reported as the logarithm base 2 of the fold change of each compound. Affected metabolic pathways were estimated using the Kyoto Encyclopedia of Genes and Genomes (KEGG) Pathway database (Kanehisa and Goto, 2000; Maridueña‐Zavala et al., 2017) with *Musa acuminata* Colla as the model organism. Multivariate data analysis was carried out using XLSTAT 2018 (Addinsoft, Paris, France) and included principal component analysis (PCA) as well as partial least squares discriminant analysis (PLS‐DA) with jacknife cross‐validation and estimation of variable importance in the projection (VIP). Metabolites with VIP values above 2 (Steinfath et al., 2010) and the differentially expressed metabolites selected by Student's *t*‐test (Cevallos‐Cevallos et al., 2009) were considered to be potential biomarkers for SV.

For SV detection, three PLS‐DA models were created using the metabolite profile of 36 samples, and each model targeted the detection of the SV, CMV, or N sample class, respectively. Each model was built by assigning scores of 1 for samples belonging to the target class and zero for the other samples, and plants with model prediction values above 0.5 were assigned to the target class. The models were then validated using the remaining 15 plants.

## RESULTS

A total of 466 metabolite peaks were detected in the GC‐MS runs, but Student's *t*‐test comparisons with N plants revealed that only 82 metabolites were differentially expressed (*P* < 0.05) in SV or CMV‐infected samples. Table 1 shows all the differentially expressed metabolites and the pathways potentially affected in SV and CMV‐infected plants.

**Table 1:** Metabolites that were differentially expressed in dwarf (SV) and cucumber mosaic virus (CMV)–infected plants compared to normal‐healthy (N) samples.^a^

Metabolite	Relative intensity^b^			Log2 FC^c^			Potential pathways
	N	SV	CMV	SV‐N	SV‐CMV	CMV‐N	
Rhamnose	0.00 ± 0.00a	0.09 ± 0.01b	0.00 ± 0.00a	>10	>10	ND	Fructose and mannose metabolism
d‐Mannitol	0.27 ± 0.05a	0.36 ± 0.07a	0.15 ± 0.01b	0.41	1.26	−0.85	Fructose and mannose metabolism
Fructose	0.13 ± 0.06a	0.79 ± 0.22b	0.00 ± 0.00a	2.57	>10	<−10	Fructose and mannose metabolism, galactose metabolism
Fructopyranose	1.44 ± 0.39a	2.30 ± 0.36a	0.00 ± 0.00b	0.68	>10	<−10	Fructose and mannose metabolism, galactose metabolism
Fructofuranose	2.38 ± 0.36a	2.96 ± 0.76a	0.00 ± 0b	0.32	>10	<−10	Fructose and mannose metabolism, galactose metabolism
Glucose	0.78 ± 0.13a	0.93 ± 0.12a	0.04 ± 0.01b	0.27	4.50	−4.24	Starch and sucrose metabolism, galactose metabolism, fructose and mannose metabolism
Mannopyranose	3.32 ± 0.81a	4.01 ± 1.18a	0.00 ± 0.00b	0.27	>10	<−10	Fructose and mannose metabolism, galactose metabolism
Sucrose	0.63 ± 0.09a	1.54 ± 0.45a	5.16 ± 0.99b	1.28	−1.75	3.03	Galactose metabolism (produced), starch and sucrose metabolism (consumed)
d‐Lactose	0.50 ± 0.06a	0.66 ± 0.09a	0.00 ± 0.00b	0.40	>10	<−10	Galactose metabolism
Maltose	0.17 ± 0.05a	0.01 ± 0.00b	0.00 ± 0.00b	−4.88	>10	<−10	Starch and sucrose metabolism
Trehalose	0.01 ± 0.01a	0.00 ± 0.00a	1.01 ± 0.15b	<−10	<−10	6.63	Starch and sucrose metabolism (consumed)
Cellobiose	0.03 ± 0.01a	0.00 ± 0.00b	0.00 ± 0.00b	<−10	ND	<−10	Starch and sucrose metabolism
Linolenic acid	1.27 ± 0.45a	0.36 ± 0.08a	0.00 ± 0.00b	−1.81	>10	<−10	Biosinthesis of unsaturated fatty acids
Hexadecanoic acid	0.14 ± 0.03a	0.37 ± 0.05b	1.27 ± 0.20c	1.42	−1.78	3.20	Consumed by fatty acid degradation, produced by fatty acid enlongation
9,12‐Octadecadienoic acid	0.12 ± 0.03a	0.09 ± 0.02a	0.00 ± 0.00b	−0.36	>10	<−10	Biosynthesis of unsaturated fatty acids
Octadecanoic acid	0.98 ± 0.14a	0.58 ± 0.12b	0.02 ± 0.01c	−0.75	4.82	−5.57	Biosynthesis of unsaturated fatty acids
Phenylalanine	0.20 ± 0.05a	0.32 ± 0.06a	0.00 ± 0.00b	0.69	>10	<−10	Biosynthesis of amino acids
Cystathionine	0.02 ± 0.01a	0.04 ± 0.01a,b	0.00 ± 0.00b	0.61	>10	<−10	Biosynthesis of amino acids
l‐Isoleucine	0.11 ± 0.02a	0.09 ± 0.02a	0.54 ± 0.04b	−0.26	−2.56	2.30	Valine, leucine, and isoleucine biosynthesis, biosynthesis of amino acids
l‐Leucine	0.14 ± 0.03a	0.11 ± 0.02a	2.29 ± 0.49b	−0.37	−4.43	4.06	Valine, leucine, and isoleucine biosynthesis, biosynthesis of amino acids
l‐Lysine	0.10 ± 0.02a	0.08 ± 0.03a	0.00 ± 0.00b	−0.41	>10	<−10	Biosynthesis of amino acids, lysine biosynthesis
l‐Asparagine	0.27 ± 0.10a	0.08 ± 0.02a	0.00 ± 0.00b	−1.70	>10	<−10	Biosynthesis of amino acids, alanine, aspartate, and glutamate metabolism
Allothreonine	0.00 ± 0.00a	0.00 ± 0.00a	0.18 ± 0.03b	<−10	<−10	6.09	Glycine, serine, and threonine metabolism
Butanoic acid	0.14 ± 0.06a	0.11 ± 0.03a	0.00 ± 0.00b	−0.32	>10	<−10	Butanoate metabolism
2‐Butenedioic acid	0.04 ± 0.01a	0.03 ± 0.01a	0.00 ± 0.00b	−0.34	>10	<−10	Butanoate metabolism
Tyramine	0.01 ± 0.00a	0.02 ± 0.00a	0.07 ± 0.01b	1.38	−2.05	3.43	Tyrosine metabolism
Stigmasterol	0.53 ± 0.13a	0.19 ± 0.06b	0.00 ± 0.00c	−1.44	>10	<−10	Steroid biosynthesis
Ribitol	0.02 ± 0.01a	0.08 ± 0.04a	0.38 ± 0.04b	2.25	−2.18	4.43	Riboflavin metabolism, pentose glucoronate interconversions
Adenosine	0.13 ± 0.03a	0.08 ± 0.02a	0.00 ± 0.00b	−0.75	>10	<−10	Purine metabolism
Ribofuranose	0.16 ± 0.04a	0.18 ± 0.06a	0.74 ± 0.09b	0.20	−2.01	2.21	Pentose phosphate pathway
Methyl galactoside	1.38 ± 0.62a	2.53 ± 0.72a	0.00 ± 0.00b	0.88	>10	<−10	NF
Methyl galactopyranoside	0.02 ± 0.01a	0.01 ± 0.00a	0.19 ± 0.02b	−1.14	−4.63	3.49	NF
Allopyranose	1.92 ± 0.78a	5.36 ± 1.55a	0.00 ± 0.00b	1.48	>10	<−10	NF
Allofuranose	0.80 ± 0.16a	1.39 ± 0.20b	3.11 ± 0.46c	0.81	−1.16	1.97	NF
l‐Threitol	0.08 ± 0.01a	0.12 ± 0.01a	1.58 ± 0.19b	0.64	−3.76	4.40	NF
Methyl glucofuranoside	0.05 ± 0.02a	0.40 ± 0.08b	0.00 ± 0.00c	3.04	>10	<−10	NF
NI1	0.03 ± 0.03a	0.24 ± 0.08b	0.00 ± 0.00a	2.97	>10	<−10	NF
Ribonic acid	0.04 ± 0.02a	0.24 ± 0.06b	0.49 ± 0.03c	2.68	−1.03	3.71	NF
NI2	0.03 ± 0.01a	0.11 ± 0.02b	0.06 ± 0.02a,b	1.90	0.89	1.01	NF
Galactopyranosyl bromide, tetraacetate	0.22 ± 0.07a	0.81 ± 0.20b	0.00 ± 0.00c	1.88	>10	<−10	NF
NI3	0.04 ± 0.01a	0.07 ± 0.02a	0.00 ± 0.00b	0.85	>10	<−10	NF
NI4	0.05 ± 0.02a	0.09 ± 0.03a,b	0.11 ± 0.01b	0.84	−0.34	1.18	NF
NI5	2.09 ± 0.56a	3.55 ± 0.85a,b	4.06 ± 0.49b	0.77	−0.19	0.96	NF
l‐Altrose	2.03 ± 0.36a	3.00 ± 0.45a	0.07 ± 0.01b	0.56	5.41	−4.84	NF
NI6	0.04 ± 0.01a	0.07 ± 0.03a,b	0.00 ± 0.00b	0.56	>10	<−10	NF
Glucopyranosiduronic acid	0.33 ± 0.04a	0.48 ± 0.04a	0.25 ± 0.03b	0.55	0.97	−0.42	NF
Propanoic acid	0.34 ± 0.04a	0.45 ± 0.08a,b	0.55 ± 0.06b	0.43	−0.28	0.71	NF
d‐glucosone	0.17 ± 0.06a	0.22 ± 0.06a	2.04 ± 0.13b	0.38	−3.23	3.60	NF
Hexopyranose	3.52 ± 1.24a	4.57 ± 0.95a	0.00 ± 0.00b	0.38	>10	<−10	NF
Docosane	0.05 ± 0.01a	0.06 ± 0.02a	0.29 ± 0.04b	0.38	−2.28	2.66	NF
Butanedioic acid	0.45 ± 0.07a	0.57 ± 0.09a	1.20 ± 0.07b	0.34	−1.08	1.42	NF
NI7	2.38 ±0.36a	2.96 ± 0.76a	0.00 ± 0.00b	0.32	>10	<−10	NF
Galacturonic acid	0.15 ± 0.03a	0.18 ± 0.02a	0.00 ± 0.00b	0.27	>10	<−10	NF
Phosphate	0.49 ± 0.08a	0.59 ± 0.07a	0.00 ± 0.00b	0.26	>10	<−10	NF
Methyl glucopyranoside	1.41 ± 0.31a	1.69 ± 0.47a	0.55 ± 0.15b	0.26	1.63	−1.37	NF
Niacin	0.06 ± 0.02a	0.06 ± 0.03a,b	0.00 ± 0.00b	0.15	>10	<−10	NF
Phosphoric acid propyl ester	0.48 ± 0.15a	0.49 ± 0.22a,b	0.11 ± 0.07b	0.05	2.21	−2.17	NF
Phenylethanolamine	0.07 ± 0.02a	0.07 ± 0.03a	0.25 ± 0.02b	−0.06	−1.81	1.75	NF
NI10	11.59 ± 1.58a	10.37 ± 1.33a	0.00 ± 0.00b	−0.16	>10	<−10	NF
7,7′,8,8′,11,11′,12,12′,15,15′‐decahydro‐carotene	0.33 ± 0.15a	0.24 ± 0.11a	0.00 ± 0.00b	−0.48	>10	<−10	NF
1‐benzopyran‐4‐one	0.31 ± 0.09a	0.22 ± 0.06a	0.00 ± 0.00b	−0.51	>10	<−10	NF
Quinic acid	0.59 ± 0.11a	0.42 ± 0.12a	0.00 ± 0.00b	−0.51	>10	<−10	NF
l‐Threonic acid	3.77 ± 0.79a	2.45 ± 0.45a,b	1.60 ± 0.39b	−0.63	0.61	−1.24	NF
NI11	1.35 ± 0.33a	0.85 ± 0.21a	0.01 ± 0.00b	−0.67	6.62	−7.30	NF
4‐O‐β‐Galactopyranosyl‐d‐mannopyranose	0.06 ± 0.02a	0.04 ± 0.00a	0.00 ± 0.00b	−0.74	>10	<−10	NF
Glyceryl‐glycoside	2.42 ± 0.39a	1.31 ± 0.19b	0.00 ± 0.00c	−0.89	>10	<−10	NF
NI12	0.12 ± 0.04a	0.06 ± 0.02a	0.00 ± 0.00b	−1.02	>10	<−10	NF
NI13	0.15 ± 0.05a	0.07 ± 0.04a,b	0.00 ± 0.00b	−1.12	>10	<−10	NF
Dihydro‐3,4‐dimethyl‐2(3H)‐furanone	0.05 ± 0.01a	0.02 ± 0.00b	0.00 ± 0.00c	−1.17	>10	<−10	NF
NI14	0.12 ± 0.05a	0.05 ± 0.02a	0.00 ± 0.00b	−1.22	>10	<−10	NF
NI15	0.34 ± 0.08a	0.14 ± 0.03b	0.00 ± 0.00c	−1.29	>10	<−10	NF
NI16	0.16 ± 0.04a	0.06 ± 0.01b	0.00 ± 0.00c	−1.39	>10	<−10	NF
Ribono‐1,4‐lactone	0.12 ± 0.02a	0.04 ± 0.01b	0.10 ± 0.03a,b	−1.45	−1.19	−0.26	NF
NI17	0.23 ± 0.05a	0.08 ± 0.02b	2.42 ± 0.32c	−1.48	−4.87	3.39	NF
3‐Penten‐2‐one	0.21 ± 0.06a	0.07 ± 0.02b	0.00 ± 0.00c	−1.65	>10	<−10	NF
NI18	0.05 ± 0.02a	0.02 ± 0.01a,b	0.00 ± 0.00b	−1.68	>10	<−10	NF
Mannobiose	4.03 ± 1.09a	1.13 ± 0.55b	15.40 ± 1.52c	−1.84	−3.77	1.93	NF
NI19	0.37 ± 0.11a	0.09 ± 0.03b	0.00 ± 0.00c	−2.03	>10	<−10	NF
NI20	0.58 ± 0.17a	0.13 ± 0.03b	0.00 ± 0.00c	−2.20	>10	<−10	NF
NI21	0.34 ± 0.15a	0.05 ± 0.01a	0.00 ± 0.00b	−2.88	>10	<−10	NF
Tricosane	0.00 ± 0.00a	0.00 ± 0.00a	0.69 ± 0.09b	ND	<−10	>10	NF
Ethyl‐d‐glucopyranoside	0.00 ± 0.00a	0.00 ± 0.00a	0.02 ± 0.00b	ND	<−10	>10	NF

Note

NF = not found; NI = not identified.

a Metabolites with zero area values were below the detection limit of the GC‐MS instrument.

b Different letters represent significant differences in the relative intensities of each metabolite when comparing N, SV, and CMV samples.

c Values represent the logarithm base 2 of the fold change (log2 FC) in metabolite intensity between two sample classes.

All SV samples were characterized by the presence of rhamnose as this metabolite was not detected in N or CMV‐infected plants. Similarly, only SV samples showed undetectable levels of allothreonine and trehalose. Significantly higher levels of nine other metabolites, including methyl glucofuranoside, ribonic acid, fructose, hexadecenoic acid, allofuranose, butanedioic acid, and glucopyranosiduronic acid, were also characteristic of SV when compared to N samples. Conversely, the levels of 13 metabolites were significantly lower in SV than N plants, including octadecanoic acid, glyceryl‐glycoside, dihydro‐3,4‐dimethyl‐2(3H)‐furanone, stigmasterol, ribono‐1,4‐lactone, 3‐penten‐2‐one, mannobiose, maltose, and cellobiose. Significant differences (*P* < 0.05) between SV and CMV‐infected samples were observed in 68 metabolites, including 47 upregulated and 21 downregulated in SV when compared to the CMV‐infected plants using Student's *t*‐tests. Among them, sugars and sugar derivatives such as rhamnose, fructose, methyl glucofuranoside, allopyranose, lactose, and galacturonic acid; amino acids including phenylalanine and l‐lysine; and organic acids like butanoic acid and 2‐butenedioic acid were among the significantly upregulated metabolites. Likewise, some sugars and sugar derivatives like ribonic acid, allofuranose, sucrose, mannobiose, and trehalose; organic acids like butanedioic and hexadecenoic acids; and amino acids such as allo‐threonine, leucine, and isoleucine were among the significantly downregulated metabolites.

Results of the Student's *t*‐tests showed significant differences (*P* < 0.05) in the levels of 76 metabolites, including 24 metabolites upregulated and 52 downregulated in CMV‐infected plants when compared to N plants. Among the upregulated metabolites, two metabolites (tricosane and ethyl‐d‐glucopyranoside) were exclusively detected in CMV samples. Other upregulated metabolites included sugars and sugar derivatives such as trehalose, ribitol, methyl galactopyranoside, sucrose, ribofuranose, allofuranose, and mannobiose; amino acids like allothreonine, leucine, and isoleucine; and organic acids such as hexadecenoic and butanedioic acids. Among the downregulated metabolites, 45 metabolites including sugars and sugar derivatives such as methyl glucofuranoside, cellobiose, methyl galactoside, fructopyranose, lactose, and galacturonic acid; amino acids like phenylalanine, l‐lysine, and asparagine; and organic acids like butanoic, 2‐butenedioic, 9,12‐octadecadienoic, and linolenic acids were only absent in plants infected with CMV. The disaccharide cellobiose was detected in N plants only.

Pathway mapping suggests a significant upregulation of the fructose and mannose metabolism in SV plants, but other metabolic pathways were significantly downregulated in the dwarf variants, including fatty acid degradation, starch and sucrose metabolism, biosynthesis of unsaturated fatty acids, and steroid biosynthesis pathways. Similarly, various pathways were significantly downregulated in CMV samples, including fructose and mannose metabolism, galactose metabolism, starch and sucrose metabolism, biosynthesis of unsaturated fatty acids, fatty acid degradation, biosynthesis of amino acids, butanoate metabolism, steroid biosynthesis, and purine metabolism, whereas other pathways were significantly upregulated, including valine, leucine, and isoleucine biosynthesis; glycine, serine, and threonine metabolism; tyrosine metabolism; and riboflavin metabolism, as well as the pentose phosphate pathway.

**Figure 1:**
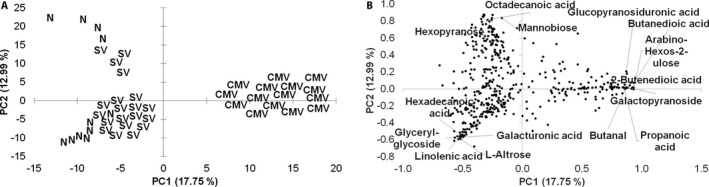
Score (A) and loading plot (B) of the principal components analysis (PCA) run on dwarf (SV), normal‐healthy (N), and cucumber mosaic virus (CMV)–infected plants. Values in parentheses represent the percentage of the variance explained by each component.

Multivariate PCA showed a clear grouping of the CMV‐infected plants and a partial grouping of the SV and N samples (Fig. 1A). Principal components (PC) 1 and 2 accounted for 30.74% of the total variation, and sample grouping occurred mostly on PC1. All CMV‐infected plants were characterized with high positive scores on PC1, whereas SV samples showed low negative score values. All N samples yielded highly negative score values in PC1. The metabolites with the highest absolute loading values in PC1 were sugars and sugar derivatives such as mannobiose, l‐altrose, galacturonic acid, hexopyranose, glucopyranosiduronic acid, d‐glucosone, and methyl galactopyranoside; organic acids including propanoic, butanedioic, octadecanoic, hexadecanoic, linolenic, and 2‐butenedioic acids; and other metabolites such as glyceryl‐glycoside and butanal (Fig. 1B).

**Figure 2:**
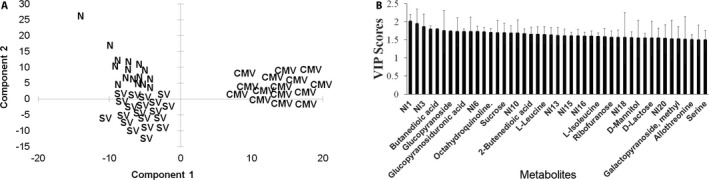
Scores (A) and variable importance in the projection (B) plots of the partial least squares discriminant analysis (PLS‐DA) run on dwarf (SV), normal‐healthy (N), and cucumber mosaic virus (CMV)–infected samples. NI = unidentified metabolites; VIP = variable importance in the projection. VIP bars are presented as mean and standard deviation of the metabolites with VIP scores of 1.5 or greater.

**Figure 3:**
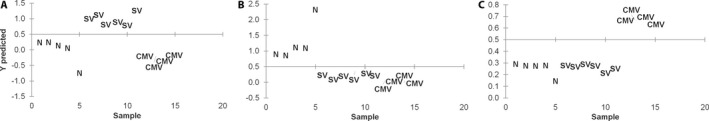
Validation of the SV (A), N (B), and CMV (C) models. SV = dwarf; N = normal‐healthy; CMV = cucumber mosaic virus–infected.

Prediction models for N, SV, and CMV sample classes were built using PLS‐DA. The models consisted of two PLS components and yielded total *R* ^2^ and *Q* ^2^ values of 0.87 and 0.71, respectively (Table 2). A 100% classification accuracy was observed in all samples used for calibration of each model, and the PLS scores plot showed a more marked grouping of SV, N, and CMV‐infected samples than PCA (Fig. 2A). The metabolites with the highest VIP scores (from 1.5 to 2.0) for the three models were sugars and sugar derivatives such as methyl glucopyranoside, glucopiranosiduronic acid, sucrose, gluconic acid, ribofuranose, mannitol, lactose, and galactopyranose; organic acids including butanedioic, 2‐butenedioic, and propanoic acids; amino acids like leucine, allo‐threonine, and serine; and the aldehyde butanal (Fig. 2B). However, none of the metabolites showed VIP values above 2 and were not considered among the potential biomarkers for SV. The potential of the three models combined for SV diagnostic was tested with 15 plants not previously used for model calibration, including five N, six SV, and four CMV‐infected plants. The models were 100% accurate in all validation tests (Fig. 3).

**Table 2:** Quality values for the PLS‐DA prediction models

Model	*R* ^2^	*Q* ^2^
CMV	0.972	0.957
N	0.799	0.578
SV	0.838	0.599
Total	0.870	0.711

Note

CMV = cucumber mosaic virus–infected plants; N = normal‐healthy plants; *Q* ^2^ = goodness of prediction obtained from the jacknife cross‐validation data; *R* ^2^ = coefficient of determination for multivariate analysis; SV = dwarf plants.

## DISCUSSION

A novel metabolite‐based approach using PLS‐DA to detect SV in banana plants is presented, and a number of metabolite biomarkers of banana SV were discovered and validated. Metabolite biomarkers can be qualitative (presence/absence) or quantitative (abundance) (Cevallos‐Cevallos et al., 2009). Quantitative biomarkers can be selected based on VIP values (Steinfath et al., 2010) or differential expression assessment by Student's *t*‐test (Cevallos‐Cevallos et al., 2009), among other methods. Because VIP values were below 2, we used the *P* values obtained by Student's *t*‐tests to select potential quantitative biomarkers.

Among the qualitative biomarkers of SV, rhamnose can be produced in banana plants through the fructose and mannose pathway; derivatives of this metabolite are required for the synthesis of important cell wall components in plants (Oka et al., 2007). Therefore, the presence of this monosaccharide in SV samples only might have contributed to the thicker pseudostem observed in the dwarf variants when compared to the N or CMV‐infected plants (Oh et al., 2007; Ferrero‐Serrano and Assmann, 2016). Trehalose—another qualitative biomarker of SV plants—is produced in plants through the dephosphorylation of trehalose‐6‐phosphate (T6P) catalyzed by T6P phosphatase (TPP) (Grennan, 2007). T6P is considered an important signaling metabolite that is involved in the regulation of plant growth (O'Hara et al., 2013). Molecules of T6P are produced by T6P synthase (TPS), and alterations in this enzyme have been shown to inhibit plant growth and trehalose production (O'Hara et al., 2013). The undetectable levels of trehalose in SV plants suggest aberrations in TPP or TPS activity resulting in the accumulation or absence of T6P, respectively. Both the absence and excess of T6P have caused stunted growth in plants (O'Hara et al., 2013) and may have contributed to the dwarfism observed in the SV samples. Further research is needed to elucidate the T6P metabolism in SV plants. Similarly, allothreonine—undetected in SV plants only—is produced by the glycine, serine, and threonine metabolism pathway, but the role of this amino acid in plant development is still unclear.

Cellobiose was the only qualitative (presence) biomarker of N plants only. This disaccharide is an intermediary metabolite in the synthesis of cellulose, a main constituent of plant cell walls (Maleki et al., 2016). The deposition of cellulose in the cell wall is essential to plant growth, and deficient cellulose production has yielded dwarfed plant mutants (Taylor, 2008). The absence of cellobiose in SV and CMV‐infected plants suggests reduced cellulose synthesis that may have contributed to the stunted growth observed in the samples.

The CMV‐infected plants showed 47 qualitative biomarkers out of which 45 were absence biomarkers (Table 1). The only CMV presence biomarkers were tricosane and ethyl‐d‐glucopyranoside, and further research is needed to establish the role of both metabolites in CMV plant infection. Reduction in levels of various metabolites has previously been reported in plant infection (Cevallos‐Cevallos et al., 2012), and an overall reduced metabolism may be occurring in CMV‐infected plants. Particularly, the absence of l‐lysine might have contributed to the stunted growth of plants infected with the virus as this metabolite has been reported to participate in reactions associated with plant growth and development (Tomar et al., 2013).

The downregulated pathways observed in SV and CMV plants, including fatty acid degradation, starch and sucrose metabolism, biosynthesis of unsaturated fatty acids, and steroid biosynthesis pathways, suggest overall inhibited assimilatory processes in the plant samples (Rojas et al., 2014). Upregulated pathways specific to CMV‐infected plants included the metabolism of various amino acids (e.g., tyrosine, leucine, isoleucine, glycine, serine, and threonine) previously reported to accumulate in plants infected with pathogens. Furthermore, the upregulation of the pentose phosphate pathway—observed in the CMV plants—has been suggested to promote the generation of reactive oxygen species and pathogenesis‐related proteins in infected plants (Rojas et al., 2014).

A clear PCA and PLS‐DA class grouping was observed CMV‐infected samples, whereas separation between SV and N samples was less obvious, suggesting similarities in the GC‐MS metabolite profile of both groups. SV and N plants were not affected by biotic stresses as the CMV‐infected plants were, and biotic stress has been shown to yield a higher number of metabolic differences than those caused by abiotic conditions in plants (Cevallos‐Cevallos et al., 2011).

PLS‐DA with validation using additional data sets was selected for the development of prediction models because this method is not sensitive to multicollinearity (Palermo et al., 2009; Worley and Powers, 2012) and has been reported to yield better class separation than other techniques such as random forest (Gromski et al., 2015). Additionally, the three classes analyzed in this experiment prevent the use of procedures designed to solve a two‐class problem, including the support vector machines algorithm. PLS‐DA has yielded similar results than the commonly used principal component–discriminant function analysis (PC‐DFA), but the ability to rank the variables responsible for class separation is greater in PLS‐DA when compared to PC‐DFA (Gromski et al., 2015). The VIP scores were used for variable selection as this technique has outperformed other methods including PLS, PCR, and Lasso regression coefficients (Palermo et al., 2009).

The PLS‐DA prediction models were able to accurately classify the samples showing dwarf SV. Metabolite‐based PLS models have been successfully used to predict phenotypic traits in plants, including the susceptibility to black spot bruising and chip quality in potato tubers (Steinfath et al., 2010). To the best of our knowledge, this is the first report of a metabolite‐based PLS‐DA model for diagnosis of dwarf bananas. The proposed methodology could be directly applied in banana plant production facilities to detect dwarf variants and discriminate SV from CMV‐infected bananas before releasing the plants to the field as SV diagnosis using only visual symptoms can be highly unreliable. A symptoms‐based preselection of plants is required before this methodology is used. Further research is needed to assess the suitability of metabolomics‐based prediction models to detect SV in banana plants at the early production stages and before the first symptoms appear.

DNA markers have also been proposed to detect SV in banana plants, but these markers have not been shown to have any association with plant phenotypic variations. For instance, the use of RAPDs was able to detect 51.4% genetic variation in banana plants produced by tissue culture (Sheidai et al., 2008), but SV was not phenotypically confirmed and no association between DNA markers and alterations of visual traits was made. Similarly, an ISSR‐based study reported about 5.0% genetic variation in micropropagated *Musa* spp., but morphological variations were not observed in the generated clones (Ray et al., 2006). The PLS‐DA model proposed in this study represents the first SV diagnosis method at the phenotypic level.

The proposed models were also able to diagnose CMV infection in banana plants at the greenhouse establishment phase. Metabolite‐based PLS models have been used for prediction of plant pathogen infections, including the presence of *Botrytis cinerea* in commercial berry groves (Hong et al., 2012). Results show the potential of metabolite‐based PLS models to detect CMV in young banana plants, but further model development is needed for CMV detection in adult plants from commercial groves, as the disease mostly occurs in banana fields where various subgroups of the pathogen can be present (Yeturu et al., 2016).

In conclusion, the metabolite profile of plants with dwarf SV offered additional insights into banana dwarfism and provided a novel alternative for the specific detection of dwarf banana variants. Because of the observed metabolome–phenotype relationships, metabolite‐based detection of dwarf SV has the potential to become a superior SV diagnosis tool when compared to molecular‐based methods. This is the first metabolite‐based characterization and detection of somaclonal variation, showing the potential of metabolomics tools to understand and selectively detect phenotypic variations in plants.

## AUTHOR CONTRIBUTIONS

J.M.C.‐C. conceived, designed, wrote, and approved the article. C.J. performed sample processing, data aquisition, and data pre‐treatment. M.G.M.‐Z. performed data validation and statistical analysis. M.J.M.‐M. performed data analysis and interpretation. D.E.O. reviewed the manuscript and obtained funding. J.A.F.‐C. designed the experiment, performed sampling, and interpreted the data.
